# Effects of environmental concentrations of caffeine on adult zebrafish behaviour: a short-term exposure scenario

**DOI:** 10.1007/s11356-023-26799-4

**Published:** 2023-04-14

**Authors:** Niedja Santos, Victor Picolo, Inês Domingues, Vitória Perillo, Rolando A.R. Villacis, Cesar Koppe Grisolia, Miguel Oliveira

**Affiliations:** 1 Centre for Environmental and Marine Studies (CESAM), Department of Biology, University of Aveiro, 3810-193 Aveiro, Portugal; 2 Department of Physiological Sciences, Institute of Biological Sciences, University of Brasilia, University Campus Darcy Ribeiro, Brasilia, DF 70910-900 Brazil; 3 Graduate Program in Molecular Pathology, Faculty of Health Sciences, University of Brasilia, University Campus Darcy Ribeiro, Brasilia, DF 70910-900 Brazil; 4 Laboratory of Toxicological Genetics, Department of Genetics and Morphology, Institute of Biological Sciences, University of Brasília, Brasília, Distrito Federal 70910-900 Brazil

**Keywords:** *Danio rerio*, Anxiety, Aggression, Sociability, Exploratory behaviour, Feeding

## Abstract

Caffeine (CAF) has been considered an emerging environmental contaminant and its presence indicator of anthropogenic contamination. This study evaluated the effects of environmental concentrations of CAF (0, 0.5, 1.5, and 300 μg. L^−1^) on the behaviour of adult zebrafish (*Danio rerio*) after 7 days of exposure. The components of feeding, locomotion, boldness (new tank test), sociability (schooling test), and aggression (mirror test) were analysed. Growth rate and weight were investigated as complementary measures. CAF (0.5, 1.5, and 300 μg. L^−1^) reduced exploratory behaviour in zebrafish, increased feeding latency time (1.5, and 300 μg. L^−1^), and decreased growth rate and fish weight (300 μg. L^−1^). CAF also induced aggressive behaviour (0.5, 1.5, and 300 μg. L^−1^) and decreased appetence to the shoal (sociability) (0.5, and 1.5 μg. L^−1^). This study showed that low doses of CAF can induce behavioural effects in zebrafish that may have significant long-term impacts on vital ecological functions.

## Introduction

Caffeine (CAF) has been considered an emerging environmental contaminant and its environmental presence indicator of anthropogenic contamination (Dafouz et al., 2018; Li et al., 2021). This is mainly due to its high consumption, whether in the form of beverage (coffee, hot chocolate, teas, among others), personal care products (shampoo, moisturiser) or pharmaceuticals, leading to its insertion into the sewage system, often ineffective in removing this substance from wastewater (Korekar et al., 2020; Quadra et al., 2020). In this context, CAF has been widely detected in treated wastewater (55–303.6 μg. L^−1^), in groundwater (0.01–0.683 μg. L^−1^), in drinking water (3.39 μg. L^−1^), in rainwater (5.4 μg. L^−1^), in rivers (0.01–49.6 μg. L^−1^), and in lakes (0.02–174 μg. L^−1^) (Biswas and Vellanki, 2021; French et al., 2015; Korekar et al., 2020; Li et al., 2020), constituting a potential threat to aquatic systems.

In the last years, several components of the individual behaviour of organisms have been used as relevant endpoints to assess effects of different types of stressors in realistic ecological scenarios. Behavioural endpoints, primarily based on the locomotory activity of organisms, have emerged as sensitive parameters, easily connected to important ecological processes (such as food-seeking, sensing predators, or conspecific interactions) on which the species’ fitness depends (Costa et al., 2020; Dell et al., 2014). Moreover, neurobehavioral function has proven to be sensitive to low concentrations of environmental contaminants (Costa et al., 2020). Tests available for studying zebrafish behaviour are set up to analyse different components of behaviour such as boldness (e.g., novel tank test) (Almeida et al., 2019; Correia et al., 2019; Neave et al., 2020; Rosa et al., 2018), aggressiveness (mirror bitting test) (Carreño Gutiérrez et al., 2018; Pham et al., 2012; Picolo et al., 2021), social interaction (shoal test) (Correia et al., 2019; Kalueff et al., 2013; Pham et al., 2012; Picolo et al., 2021), interest in food (feeding test) (Almeida et al., 2019; Correia et al., 2019; Domingues et al., 2016; Santos et al., 2018), and the ability to learn (e.g., sound stimuli test) (Bhandiwad et al., 2013; Bretschneider et al., 2013; Shafiei Sabet et al., 2015). Fish behavioural responses have been used in (eco)toxicology and pharmacology studies as a proxy to anxiety-like responses and as an indicator of exposure to neuroactive compounds (Cameron and Schoenfeld, 2018; De Farias et al., 2021).

CAF, a central nervous system stimulant, is known to induce a biphasic effect in animals. In mice (exposed to CAF from 6,250 to 100,000 μg. kg^−1^) and fish (exposed to CAF from 500 to 75,000 μg. L^−1^), CAF increased locomotion and reduced anxiety at low concentrations (mice at 6,250 μg.kg^−1^ and fish at 10,000 and 25,000 μg. L^−1^), and decreased activity and induced anxiety-like behaviour at higher concentrations (mice at 100,000 μg. kg^−1^; and fish at 50,000 and 100,000 μg. L^−1^) (El Yacoubi et al., 2000; Santos et al., 2017). In this sense, it has been described that the different behavioural responses of zebrafish after exposure to CAF result from the greater interaction of CAF with a specific adenosine receptor. CAF-induced blockage of type A1 adenosine receptors increases anxiety and autonomic excitation, while A2 blockage induces an increase in locomotion activity (Maximino et al., 2010). In rats, this difference in the mode of action of CAF has also been associated with the type of receptor with which CAF interacts with greater intensity. For example, an increase in locomotor activity described in rats exposed to low doses of CAF (6250 μg. kg^−1^) occurs through the interaction of CAF with A2A-type adenosine receptors (El Yacoubi et al., 2000). The depressant effect is more frequently detected at higher doses (100,000 μg. kg^−1^) and results from A1 receptor blockage (El Yacoubi et al., 2000). In addition, blocking A2A type receptors decreases food-seeking in rats (Micioni Di Bonaventura et al., 2012). Sweeney et al. (2016) observed that CAF (6000 and 12,000 μg. kg^−1^) increases food intake in rats after 2 h of exposure. However, after 5 h of exposure, only rats exposed to 6000 μg. kg^−1^ of CAF maintain the highest food intake.

In the literature, there are numerous studies on the harmful effects of CAF on fish, describing changes on biochemical and behavioural patterns of larvae (De Farias et al., 2021; Pruvot et al., 2012) and adult fish (Ladu et al., 2015; Neri et al., 2019; Rosa et al., 2018; Santos et al., 2017). However, most of these studies assessed effects of high concentrations of CAF, which are not environmentally relevant. Therefore, there is the need to perform studies with CAF in the range of ng. L^−1^ to μg. L^−1^, as detected in the aquatic environment. Thus, the present work aimed at evaluating the effects of CAF in adult zebrafish within a range of concentrations with environmental relevance. For this, a series of well-stablished tests were performed to evaluate several behavioural parameters, including locomotor activity, vertical exploration in a new environment, aggression, social behaviour, response to sound stimuli, and eating behaviour. Results will contribute to clarify the effects of CAF on fish behaviour and the potential consequences of CAF as an environmental contaminant for non-target aquatic organisms.

## Materials and methods

### Chemical

Caffeine (CAF) (CAS number: 58-08-2) was purchased from Sigma-Aldrich (Brazil). Stock solutions of CAF were prepared by dissolution in zebrafish culture water. Test solutions were prepared by diluting the stocks.

### Test organisms

Zebrafish were raised and maintained in a recirculation system (ZebTec - Tecniplast, Italy) with a photoperiod cycle of 12:12 h (light: dark) at the University of Brasilia (Brazil). The water parameters were strictly controlled: temperature was maintained at 27.0 ± 1°C, conductivity at 650 ± 100 μS/cm, pH at 7.0 ± 0.5, and dissolved oxygen saturation ≥95%. This culture water was also used to prepare the stock and exposure solutions in all the performed tests.

### Experimental design

Adult zebrafish (AB; 5 months) were exposed to CAF (0, 0.5, 1.5, and 300 μg. L^−1^) for 7 days. The selected concentrations were based on CAF levels reported in surface waters (from 0.68 to 1.6 μg. L^−1^) (Nödler et al., 2014; Siegener and Chen, 2002) and in effluents (303 μg. L^−1^) (Korekar et al., 2020). The exposure period was based on previous studies reporting CAF-induced behaviour alterations in zebrafish larvae (e.g., total swimming time and the distance travelled) exposed for 7 days to 0.6 μg. L^−1^ CAF which is also an environmental relevant concentration (De Farias et al., 2021).

The test was conducted in accordance with OECD 215 guidelines (OECD, 2000). Fish (240) were randomly distributed in twelve tanks filled with 4 L of solution (20 fish per tank—10 male and 10 female and 3 tanks per treatment). The test solutions were renewed every 2 days (Lam et al., 2004). During the experiment, fish were fed once a day with Tetra ColorBits fish food (EUA) corresponding to 2% of the fish weight in the tank and *Artemia naupilii*. Photoperiod and the physicochemical parameters of the water were kept like those in the cultivation conditions (‘Test organisms’).

At the beginning of the exposure (0 h) and after the 7 days of exposure, fish were weighed, and the pseudo-specific growth rate (*r*) was determined following the OECD guideline (OECD, 2000). A total of 12 fish per treatment were used for each behaviour analysis, performed after the 7 days of exposure period (‘Behaviour tests’). Different sets of animals were used for each test, to minimise the impact of stress of handling, conditioning or habituation on the tested endpoints. Behavioural activities were recorded for further analysis, by an analyst blinded to the experimental condition of the fish.

### Behaviour tests

#### Feeding test

In the feeding test, fish from each treatment (*n*=12) were transferred individually to an aquarium (18 cm length × 12 cm height × 8 cm width). After the acclimation period (1 min), a portion of food (Tetra ColorBits food) corresponding to 2% of the fish body weight was provided when animals were at the bottom of the tank. The acclimatisation time was determined based on a preliminary test that indicated no significant differences between 1 and 3 min of acclimatisation, a period used by other researchers (Almeida et al., 2019). The time fish took for the first bite (ti) and the time for total food intake (tf) was measured with the help of a stopwatch. The test had a maximum duration of 10 min per fish (Chollett et al., 2014; Domingues et al., 2016).

#### Mirror biting test

In the aggression, test fish (*n*=12) were individually placed in an aquarium (20 cm length × 18 cm height × 15.5 cm width), containing a mirror (15 cm) in one side of it (Pham et al., 2012). A video camera (acA1300-200um - Basler ace) placed approximately 1 m above the tank was used to monitor the location and attempts to contact the mirror—representative of aggression. Following the test time established by Picolo et al. (2021) as soon as the individual fish were introduced in the middle of the aquarium, a 6-min session was recorded with the help of the video-tracking system ANY-maze™ (Stoelting, CO, USA), at 30 frames per second. In the analysis, the aquarium was virtually divided in three distinct zones: zone of contact with the mirror (0.5 cm—zone 1), zone of approach (2.5 cm—zone 2), zone distant from the mirror (17 cm—zone 3). The following endpoints were analysed: time until the first approach to the mirror to attack, a “conspecific” represented by the image itself (latency), number of times the fish bit the mirror (mirror biting frequency), and the duration of the bite on the mirror (mirror biting duration). All parameters evaluated in this study corresponded to the time of fish activity only in zone 1, no parameters were analysed in zones 2 and 3.

#### Sound stimuli test

In adult zebrafish, a fast escape response can be observed within 13 ms after a stimulus (Kimmel et al., 1974) which is characterised by a contraction of the body in a C-shaped movement (Neo et al., 2015). Therefore, in this study, a sound stimuli test was devised to analyse the effect of CAF on the response to a stressful stimulus assessing the startle response (C-shape movement) based on the works of Neo et al. (2015) and Higgs et al. (2001). Briefly, 12 fish from each treatment were individually transferred to a new aquarium (20 cm length × 18 cm height × 15.5 cm width). The sound stimulus was generated by two speakers (CB4500, Blaupunkt, Germany) placed below the tank (one at each end of the aquarium), connected to an amplifier. The stimuli were of regular intermittent type—since intermittent stimuli can induce stronger effects compared to continuous stimuli (Neo et al., 2015), and consisted of the reproduction of sound pulses lasting 3s, interspersed with intervals of 27s of silence, based on the study conducted by Neo et al. (2015). The sound pressure used was of the soft type (112 ± 1 dB re 1 μPa) (Neo et al., 2015) and the frequency used was 800 Hz—frequency of high auditory sensitivity in adult zebrafish according to Higgs et al. (2001). The test lasted 3 min, being the first minute of acclimatation. Fish acclimatisation time was established based on a preliminary test that demonstrated no significant differences in exploratory behaviour between the 1 and 3 min, a period of acclimation used by other researchers (Almeida et al., 2019). Behaviour was recorded with an infrared camera (acA1300-200um - Basler ace) for further analysis. The sound stimuli lead to the activations of the motor neurons, which fire synchronously, causing the fish to bend in a characteristic ‘C’ shape away from the stimulus direction. This movement is easy to seek and differentiate from a normal swimming movement (Bhandiwad et al., 2013). The number of times fish bent into a C-shape in response to the sound stimulus was manually counted every 30 s.

#### Novel tank test

The novel tank test was performed to analyse locomotor/exploratory activity of zebrafish in a new environment (Costa et al., 2020). Fish were individually (*n*=12) introduced in a new aquarium (20 cm length × 18 cm height × 15.5 cm width), containing a 12-cm water column. Behaviour was recorded for 6 min according to Picolo et al. (2021) with an infrared camera (acA1300-200um - Basler ace), placed in front of the aquarium (18.5 cm distant). The aquarium was virtually divided into three areas—bottom, middle, and top, to discriminate vertical swimming activity. The videos recorded were analysed using video-tracking software (ANY-maze™, Stoelting CO, USA) at 30 frames per seconds. The following parameters were evaluated: total distance travelled, absolute turn angle, average speed, time spent in each area, distance travelled per area, and vertical transitions (number of entries per area).

#### Social test

The assay was performed accordingly to Calcagno et al. (2016). Briefly, the test was carried out with two aquariums of different sizes: a large aquarium (20 cm length × 18 cm height × 15.5 cm width), where the tested fish (*n*=12) were placed, and adjacent to it a small aquarium (10.5 cm length × 10.5 cm width) containing a school of 6 zebrafish. The tested fish were introduced individually in the centre of the large aquarium, and their movement was recorded, for 6 min (Correia et al., 2019; Picolo et al., 2021), with a camera (acA1300-200um - Basler ace) placed 30 cm away from the aquarium. Subsequently, the videos were analysed with the ANY-maze video-tracking software (ANY-maze™, Stoelting CO, USA) at 30 frames per seconds. The large aquarium was virtually divided into three zones of equal size: zone close to the group—zone 1; middle zone—zone 2; and the furthest zone from the school—zone 3. The following parameters were analysed: swimming distance, swimming time, number of entries in each zone.

### Statistical analysis

Pseudo-specific growth rate (*r*) was calculated according to the following equation (OECD 2000):

$$r:\frac{\log e\ w2-\overline{\log e\ w1}}{t2-t1}\times 100$$

*r*: “pseudo” specific growth rate

*w*1: weight of each fish at the beginning of the test

*w*2: weight of each fish at the end of the test

$\overline{\log e\ w1}$= average of the logarithms of the values *w*1 for the fish in the tank at the start of the experiment.

$\overline{\log e\ w2}$== logarithm of the weight of an individual fish at the end of the experiment.

*t*1, *t*2 = time (days) at start and end of study period.

All analyses were performed using Sigma plot V.12.5 (SysStat software Inc., CA, USA) statistical package. Data sets of the final weight, social test, and novel tank test passed the normality (Shapiro-Wilk) and homogeneity of variance (Brown-Forsythe test) tests, and thus, a one-way ANOVA followed by Dunnett’s multi-comparison test was used to discriminate differences towards control. Data sets of pseudo-specific growth rate, feeding test, locomotion activity, and mirror biting test did not follow a normal distribution, and thus were analysed with a one-way ANOVA on ranks (or Kruskal-Wallis), followed by Dunn’s multi-comparison test. The sound test was analysed by a two-way ANOVA (concentration and stimulus were set as factors) followed by Holm-Sidak multi-comparison test. The *H* and *p* values were reported for non-parametric data, while the *F* and *p* values were presented for parametric data. Differences were considered statistically significant when *p* <0.05.

## Results

### Mortality, weight, and growth rate

During the exposure period, there was no mortality in any treatment. Considering that the same number of males and females were used, sex was not considered as a factor in data analysis.

**Fig. 1:**
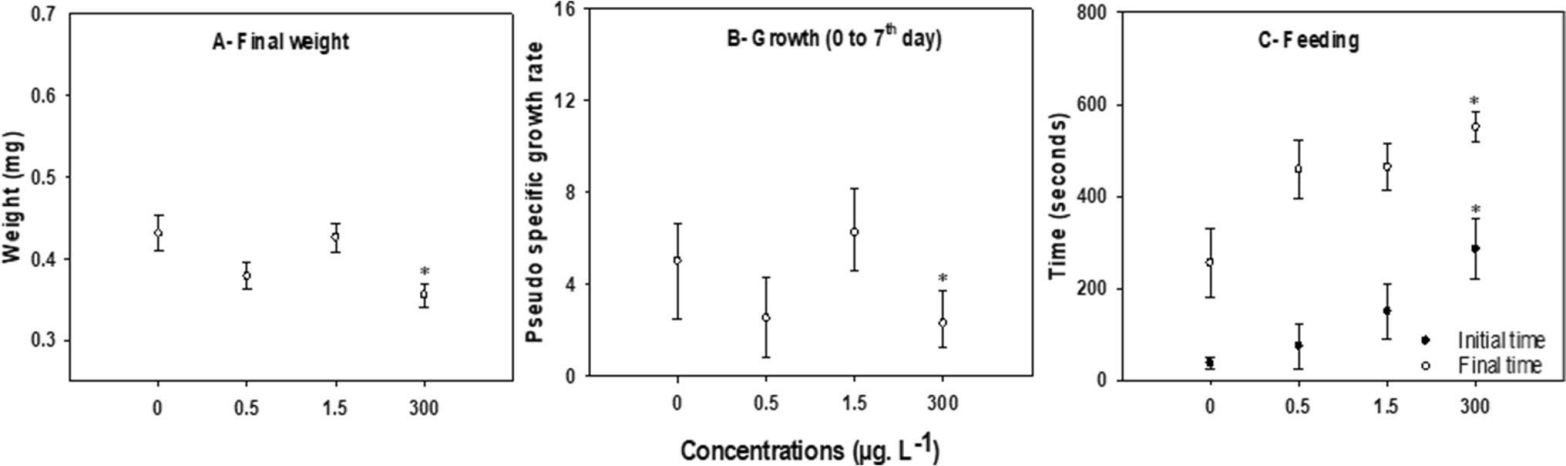
Effects of 7 days exposure to caffeine (CAF) (0, 0.5, 1.5, and 300 μg. L^−1^) on adult *Danio rerio* (*n*=12). **A** Fish weight; **B** pseudo-specific growth rates (*r*); **C** feeding behaviour (presented as time to first feeding action (black squares) and time to total food intake (grey squares). Data are expressed as mean ± standard error in graphs A and C and as median ± interquartile range in graph B. Asterisks (*) represent significant differences (*p*<0.05) from the control

The final weight (*F*=4.410, *p*≤ 0.008, Fig. 1A) and ‘pseudo’ specific growth rate (*H*= 19.587, *p*≤ 0.001, Fig. 1B) of fish exposed to the highest concentration of CAF (300 μg. L^−1^) were significantly lower than control (83% and 56% of the control group, respectively).

### Behaviour assays

#### Feeding behaviour

CAF significantly affected feeding behaviour. There was a dose-dependent increase in the time for the first feeding action (latency time) (*H*= 8.006, *p*≤ 0.046) and in the time for total food intake (Fig. 1C) (H= 10.929, *p*≤ 0.012) in the exposed fish. Latency time increased significantly in fish exposed to 300 μg. L^−1^ CAF, reaching 763% of the control level. However, no significant effects were found on the time spent eating (tf-ti) (*H*= 3.299, *p*≤ 0.348) (data not shown).

#### Novel tank test and locomotor activity

**Fig. 2:**
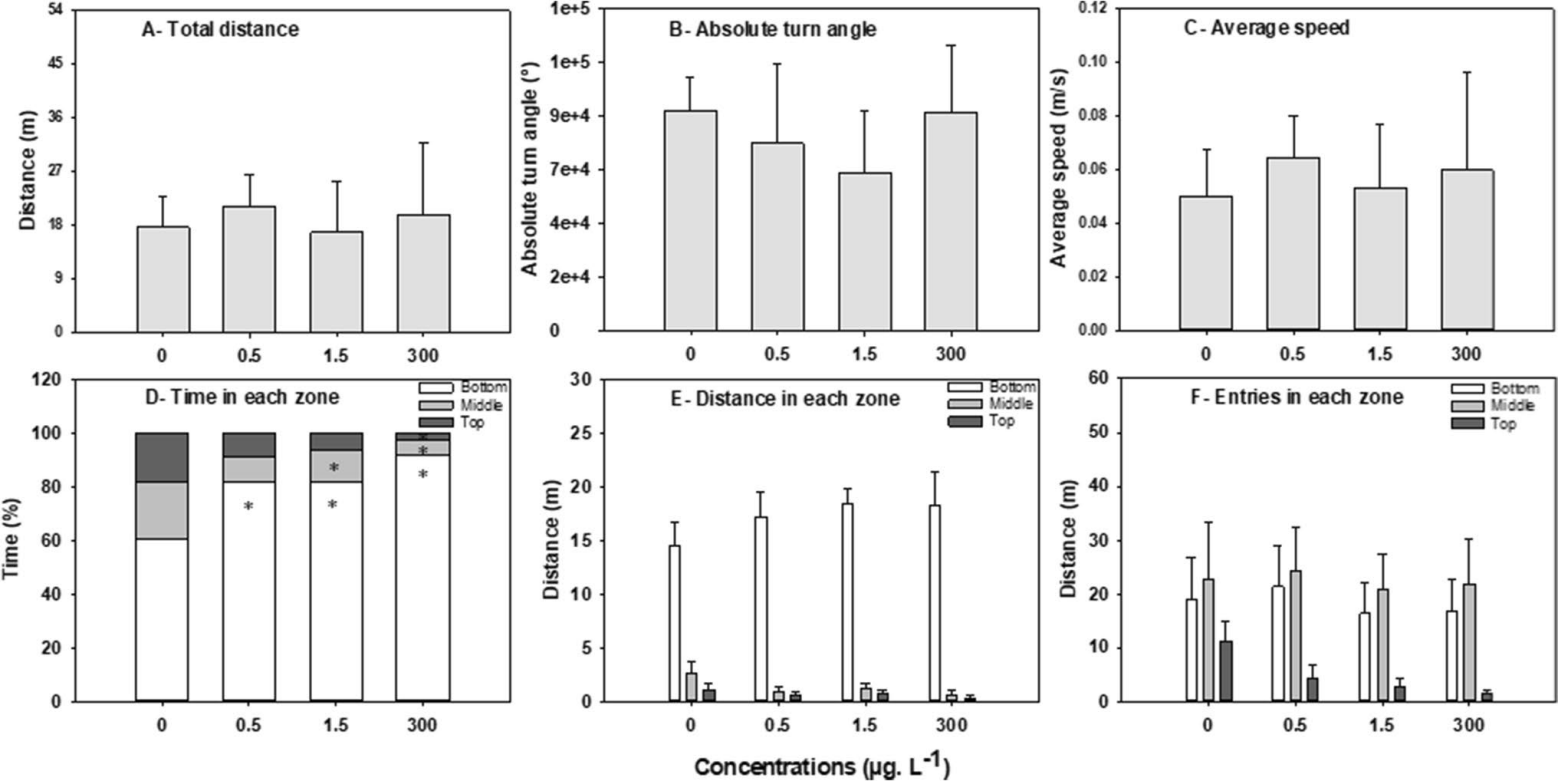
Effects of 7 days of exposure to caffeine (CAF) (0, 0.5, 1.5, and 300 μg. L^−1^) on adult *Danio rerio* locomotor activity, exploratory behaviour, and anxiety-like behaviour (novel tank) (*n*=12). **A** Distance travelled; **B** absolute turning angles; **C** average speed; **D** time (in percentage) spent swimming in the bottom (white bar), middle (light grey bar), and top (dark grey bar) zones of the aquarium; **E** distance travelled in the bottom (white bar), middle (light grey bar) and top (dark grey bar) zone of the aquarium; **F** number of entries in each zone. Data are expressed as mean ± standard error in graphs D, E, and F; and, as median ± interquartile range in graphs A, B, and C. Asterisks (*) represent significant differences (*p*<0.05) from the control

Effects of CAF on zebrafish locomotor activity are displayed in Fig. 2. Exposure to CAF did not lead to significant changes in the total distance travelled (*H*= 1.638, *p*≤ 0.651, Fig. 2A), absolute turning angles (*H* = 4.137, *p*≤ 0.247, Fig. 2B) or the average swimming speed of fish (*H* = 2.631, *p*≤ 0.452, Fig. 2C).

Regarding the exploratory appetence, when compared to the control group, fish exposed to CAF (0.5, 1.5, and 300 μg. L^−1^) spent significantly more time at the bottom (81.9%, 81.7%, and 91.8% of the time respectively, while the control group spent 60.6% of the time in the bottom) (*F*= 11.363, *p*≤ 0.010); spent less time at the middle zone—12.1% and 5.4% of the time (at 1.5, and 300 μg. L^−1^, respectively) versus 21.2% in the control group (*F*= 48.821, *p*≤ 0.001); and at the top of the tank—2.8% at 300 μg. L^−1^ (*F*= 23.073, *p*≤ 0.001), versus 18.1% of the time in the control group (Fig. 2D). Exposed fish (0.5, 1.5, and 300 μg. L^−1^) travelled greater distances at the bottom zone of the aquarium (*F*= 2.934, *p*≤ 0.402, Fig. 2E) and presented fewer entries in the top zone of the aquarium (*F*= 2.243, *p*≤ 0.524, Fig. 2F), but no significant differences to control group were detected.

#### Mirror biting test

**Fig. 3:**
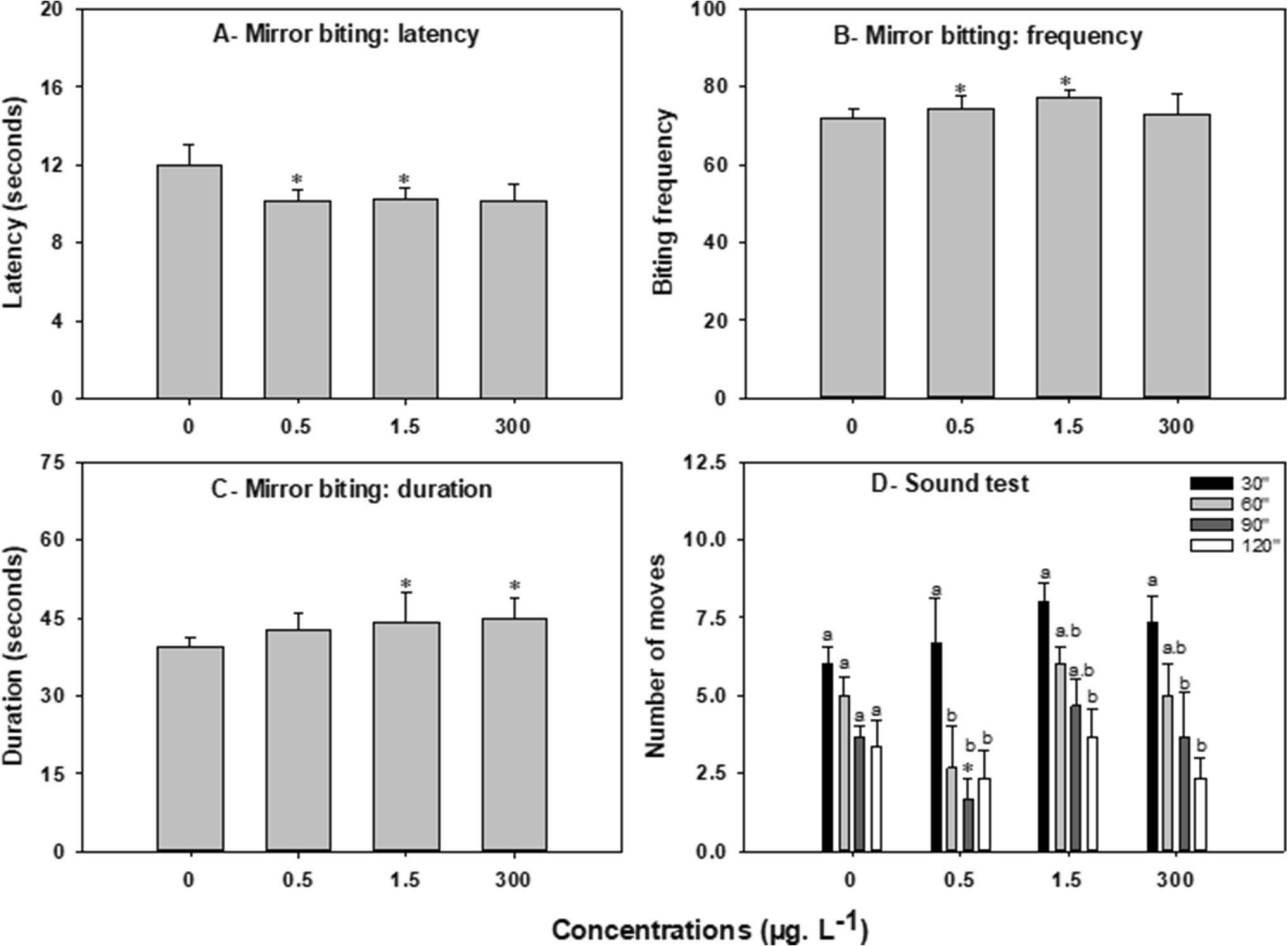
Effects of 7 days exposure to caffeine (CAF) (0, 0.5, 1.5, and 300 μg. L^−1^) on adult *Danio rerio* aggressive behaviour (mirror biting) (*n*=12) and learning (sound test) (*n*=12). **A** Latency for the first contact with the mirror; **B** frequency of contact; and **C** duration of contact; **D** number of responses to sound stimulus at 30” (black bar), at 60” (light grey bar), at 90” (dark grey bar), and at 120” (white bar). Mirror biting (A, B, and C) results are expressed as median ± interquartile range, and sound (**D**) as mean ± standard error. Asterisks (*) represent significant differences (*p*<0.05) from the control

Exposure to CAF-induced aggressive behaviour in zebrafish (Fig. 3). In the mirror biting test, exposed fish significantly decreased the time of latency to the first approach to the mirror at concentrations 0.5 and 1.5 μg. L^−1^ (*H*= 9,029, *p*≤ 0,029) (81% and 76% of the control level respectively; Fig. 3A). The frequency of mirror biting was significantly increased at 0.5 and 1.5 μg. L^−1^ (*H*= 7.904, *p*≤ 0.048) (110% and 108% of the control levels, respectively; Fig. 3B). The biting duration was significantly increased at 1.5 and 300 μg. L^−1^ (*H*= 7,999, *p*≤ 0,046) (120% and 126%, respectively; Fig. 3C).

#### Sound stimuli test

The statistical analysis revealed a low effect of CAF on the responses to sound stimulus (Fig. 3D). Differences to control were only detected in organisms exposed to 0.5 μg. L^−1^ in the 90-s period assessment (number of responses to the sound was 55% lower than control) (*F*= 4.101, *p*≤ 0.014). However, within each CAF treatment, the pattern of response along time varied considerably. In the control, the number of moves measured after consecutive stimuli did not vary significantly; while in the CAF concentrations, the number of moves after the first sound stimulus (30 s) was always higher than the number of moves after the last stimulus (120 s) (*F*= 16.067, *p*≤ 0.001).

#### Social test

**Fig. 4:**
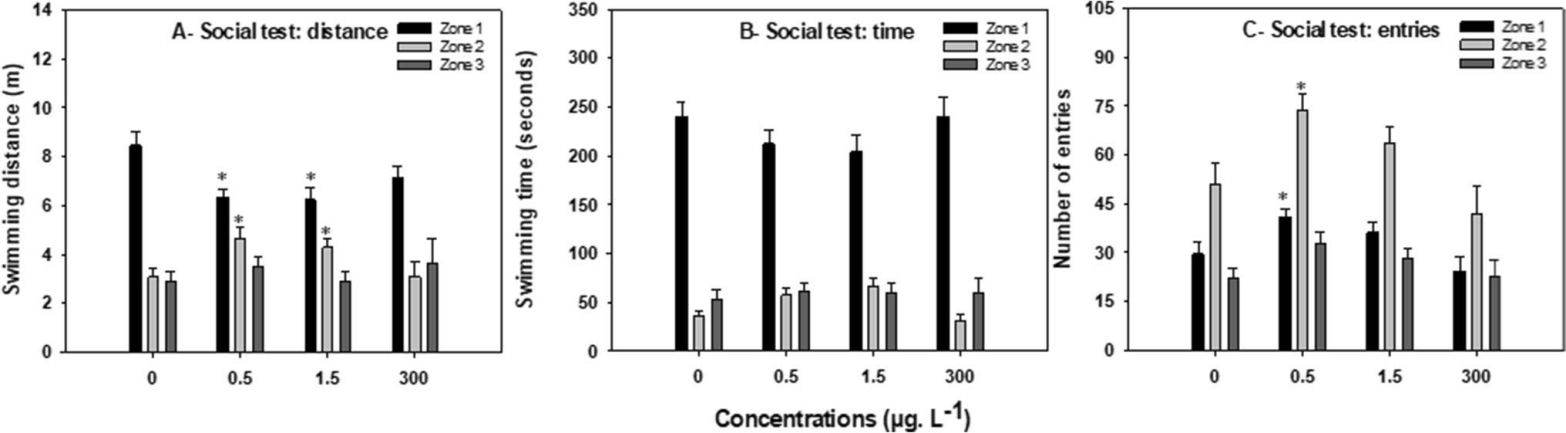
Effects of 7 days exposure to caffeine (CAF) (0, 0.5, 1.5, and 300 μg. L^−1^) on adult *Danio rerio* social behaviour (*n*=12). **A** Distance travelled in the three zones of the aquarium (zone 1—area closer to the school (black bar), zone 2—intermediate area (light grey bar), and zone 3—area farthest from the school (dark grey bar), **B** time spent in the three zones of the aquarium (zone 1, zone 2, and zone 3); **C** number of transitions/entries per aquarium zone. Results are expressed as mean ± standard error. Asterisks (*) represent significant differences (*p*<0.05) from the control

Social behaviour was affected by exposure to CAF (Fig. 4). Fish exposed to the lowest concentrations of CAF (0.5, and 1.5 μg. L^−1^) significantly decreased the distance travelled in the zone close to the school (zone 1) (75%, and 74% of the control levels respectively) (*F*= 10.228; *p*≤ 0.017) and increased the distance travelled in the intermediate zone (zone 2) (152% and 140%, respectively; Fig. 4A) (*F*= 8.722; *p*≤ 0.049). These differences were, however, not reflected in the time spent in the different zones (Fig. 4B). Additionally, fish exposed to 0.5 μg. L^−1^ showed an increment of 39% and 45% in the number of entries in the zones 1 (*F*= 4.492, *p*≤ 0.008) and 2 (*F*= 8.886, *p*≤ 0.031) respectively when compared to the control group (Fig. 4C).

## Discussion

CAF is a central nervous system stimulant known to induce behavioural changes in different organisms (e.g., mice and fish) (El Yacoubi et al., 2000; Santos et al., 2017).

The novel tank test was used to evaluate the exploratory behaviour of the zebrafish or boldness when inserted in a new environment. Typically, zebrafish tend to stay at the bottom of a new aquarium (new environment) before starting to explore the entire water column, a behaviour sensitive to anxiolytic or anxiogenic treatments (Costa et al., 2020). In this study, a significant increase in the time spent at the bottom of the aquarium and consequently a reduction in the time spent in the middle and top layers were observed in fish exposed to CAF, suggesting a decreased boldness or exploratory appetence. This impairment is considered a sign of anxiety in fish and represents a decrease in risk-taking (or an increase in protective/cautious behaviour), compatible with CAF-induced neurobehavioural responses reported in other studies (Neri et al., 2019; Rosa et al., 2018). For example, wild-type adult zebrafish exposed to CAF (50,000 and 100,000 μg. L^−1^) for 15 min showed a reduction in the vertical transitions and in the time spent at the top of the aquarium, while *leopard*-type zebrafish exposed to CAF (100,000 and 200,000 μg. L^−1^) for 15 min reduced transitions from bottom to the top and increased latency for entering the top without affecting the time spent in the top area (Rosa et al., 2018). Based on the time that fish spend in different zones of the aquarium, this study suggests that CAF concentrations decrease zebrafish exploratory behavior. The study demonstrates that low CAF concentrations (0.5, and 1.5 μg. L^−1^) induce less effects (e.g., altering vertical exploration behavior to a lesser extent than high concentrations). Data suggest that CAF decreased boldness or exploratory behavior in fish. Very often, anxiety behavior is manifested by an increase of the absolute turn angles (a measure of erratic swimming) and by the increase of the swimming speed (a measure of hyperactivity). Although CAF acts as a stimulant; in this study, changes in the total distance travelled, absolute turn angle, and average speed were not observed, suggesting that locomotion was not the most sensitive parameter in assessing CAF short-term toxicity at low concentrations in adult zebrafish or that CAF effects on locomotion are dependent on specific adenosine receptors (Ladu et al., 2015). CAF is a non-selective antagonist of the A1 and A2 adenosine receptors. Therefore, the effects of CAF on behaviour depend on which receptor is blocked. In mice, increased locomotor activity after exposure to CAF (62,5000; 12,500; 25,000; 50,000; and 100,000 μg. kg^−1^) is associated with blockade of A2A-like receptors, whereas a reduction in locomotion is associated with blockade of A1-like receptors (El Yacoubi et al., 2000). In zebrafish, A1 receptor blockade induces anxiety, while A2 blockade induces an increase in swimming activity (Ladu et al., 2015; Maximino et al., 2011).

The feeding behaviour has often been used as a biomarker able to provide ecological relevant indications because it directly interferes with the organism’s growth, reproductive status, and survival (Domingues et al., 2016). In this study, the increase in latency time to approach food, observed in fish exposed to CAF, may be due to a decrease in risk-taking behaviour or decreased exploratory behaviour as observed in the novel tank test. Alternatively, there is evidence that CAF can drastically reduce appetite and increase the satiety hormone PYY in humans (Greenberg and Geliebter, 2012) explaining the lack of interest in the food and higher latency time. However, CAF did not change the time spent for the total consumption of food, suggesting that feeding itself was not impaired and that the higher latency to feeding is associated with anxiety behaviour and reduced risk-taking. In line with these findings, the loss of weight observed in fish exposed to 300 μg. L^−1^ is not due to feeding impairment but is more likely related to changes in the metabolism needed to cope with chemical stress. To our knowledge, this is the first study analysing the eating behaviour of zebrafish after exposure to CAF; further studies are needed to clarify the mechanisms involved. A change in feeding behaviour, growth rate, and exploratory behaviour was also observed in zebrafish exposed to the anthelmintic ivermectin. The authors of the study suggested that these behavioural changes resemble those described for anticholinergic agents (Domingues et al., 2016). Interestingly, it is known that CAF can interfere with acetylcholine-based transmissions (Pohanka and Dobes, 2013). Inhibition of acetylcholinesterase generates an accumulation of acetylcholine in neuromuscular junctions, inducing overstimulation of muscarinic and nicotinic receptors, which consequently induces neurotoxicity (Adeyinka and Kondamudi, 2021; Colovic et al., 2013; Korabecny and Soukup, 2021; Punga and Stålberg, 2009). A recent study demonstrated that zebrafish embryos/larvae exposed to CAF (from 1.5 to 168 hpf) showed a significant inhibition of AChE activity (LOEC_168hpf_= 8800 μg. L^−1^) which authors suggested can be related to a reduction in total distance travelled and swimming time (LOEC _168hpf_= 6000 μg. L^−1^) (De Farias et al., 2021).

Zebrafish are highly social, swimming in small schools (Suriyampola et al., 2016). In the laboratory, social behaviour is determined by the preference to stay close to conspecifics (Ogi et al., 2021). In the present study, fish exposed to low concentrations of CAF (0.5, and 1.5 μg. L^−1^) decreased the distance moved near the school but increased the number of times they entered this zone, suggesting that CAF modulates the individual response to the presence of the school. Baggio et al. (2018) suggested that a decrease in the social interaction could be the result of increased anxiety. CAF-induced anxiety behaviour is well described in zebrafish (Alia and Petrunich-Rutherford, 2019; Rosa et al., 2018) and is consistent with the anxiogenic properties of CAF commonly attributed to non-selective antagonism of adenosine receptors (El Yacoubi et al., 2000; Rogers et al., 2010). However, in the works of Ladu et al. (2015), a robust tendency to form a school in zebrafish exposed to CAF (25,000; and 50,000 μg. L^−1^) for 20 min was observed. On the other hand, another study with adult zebrafish exposed for 1 h to CAF (25,000; 50,000; and 70,000 μg. L^−1^) demonstrated that when exposed fish swam with a specific group of four untreated fish, it swam farthest from to the group than the unexposed fish; therefore, the authors concluded that the lowest dose tested of CAF (25,000 μg. L^−1^) induced a leading role in the treated fish (Neri et al., 2019). Baggio et al. (2018) hypothesised that social behaviour decrease is connected to anxious activity. However, in the present study, fish exposed to 300 μg. L^−1^, which exhibited the highest reduction in exploratory behaviour (typical anxiety behaviour), displayed no decrease in social behaviour. Based on the findings by Neri et al. (2019) that demonstrated that CAF (25,000 μg. L^−1^) promotes a leadership role in fish, and on the data obtained in the social test in this study, namely the decrease in the distance travelled in zone 1 and increase in the distance travelled in zone 2, it is proposed that low CAF concentrations (0.5 μg. L^−1^) may make fish less group dependent, i.e., less sociable.

As a social species, interaction between zebrafish can involve aggressive responses due to territory domain behaviour (Gerlai et al., 2000; Moretz et al., 2007). Therefore, the image reflected in the mirror can be interpreted by zebrafish as a conspecific or a rival which can trigger attraction or aggression (Gerlai et al., 2000). In this study, the latency for the first approach to the mirror was reduced and number of contacts with the mirror increased in organisms treated with 0.5 and 1.5 μg. L^−1^, suggesting that low concentrations of CAF trigger an immediate active response of zebrafish when in the presence of a ‘rival’. This may be explained by the effects of CAF on adenosine receptors present in dopamine-rich brain regions (e.g., striatum and nucleus accumbens) which involve alertness and executive control of visual attention (Brunyé et al., 2010). Contrary to our results, Gutiérrez et al. (2020) demonstrated that a 30 min exposure to 19,420 μg. L^−1^ CAF decreases mirror-induced aggression in juveniles. The authors suggest that this decrease reflects a decrease in fish attention. In a 5-option reaction task series, fish exposed to 9710 μg. L^−1^ CAF showed a decrease in attention demonstrated by the reduction in the number of correct responses, while an increase in impulsive responses was detected in fish exposed from 9710 to 19,420 μg. L^−1^ of CAF (Gutiérrez et al., 2020). The differences between the results of the present study and of Gutiérrez et al. (2020) may be attributed to the known biphasic effect of CAF. CAF at low concentrations (0.6 μg. L^−1^) increases the distance travelled by zebrafish larvae while inhibiting exploratory behaviour at high doses (50,000 μg. L^−1^) (De Farias et al., 2021). In this sense, it is proposed based on the parameter latency time for approaching the mirror that CAF can improve attention. Furthermore, based on the parameter’s frequency and duration of contact, it is suggested that CAF increases aggressive behaviour at low concentrations, as those evaluated in this study (0.5, 1.5, and 300 μg. L^−1^), while it may act in the opposite way at high concentrations decreasing attention and aggressive behaviour as observed by Gutiérrez et al. (2020) at 9710 to 19,420 μg. L^−1^).

The startle response after a sound stimulus is commonly studied in zebrafish larvae (e.g., Bretschneider et al. (2013), Bhandiwad et al. (2013), and Privat et al. (2019); however, studies on adult zebrafish have also been conducted, allowing the analysis of their auditory capacity (Bang et al., 2002). The acoustic startle response is produced after a sudden loud sound and is generated by a neural circuit initiated by a single action potential in Mauthner cells, which, after a stimulus, causes the fish to bend into a ‘C’ shape (Fetcho, 1991; Ogino et al., 2019; Roberts et al., 2011). Adult zebrafish also respond to the sound stimulus with a C-startle scape response (Bang et al., 2002; Eddins et al., 2010; Kalueff et al., 2013; Neo et al., 2015; Park et al., 2018) which has been used as model for neurobiological investigations of simple forms of learning and memory (Ogino et al., 2019) and that can be modulated by different drugs, including those that target dopaminergic and glutamatergic signalling (Banono and Esguerra, 2020; Best et al., 2008). Zebrafish larvae that have been exposed for 24 h to drugs like rolipram (PDE4 inhibitor), donepezil (AChE inhibitor), and memantine (N-methyl-D-aspartic acid receptor antagonist) presented increases in acoustic startle response which may represent an improvement in the alertness or awareness of fish (Best et al., 2008). In the current study, zebrafish exposed to CAF reduced the number of responses to the sound stimulus along the repetitions suggesting desensitisation (sensory adaptation) or learning. The desensitisation/adaptation is generated by the entry of glycinergic inhibitors into the Mauthner cell; however, the molecular basis involved in the glycinergic transmission process is still not well defined (Ogino et al., 2019). On the other hand, sound stimulation also acts on the dorsal raphe nucleus in serotonergic neurons; however, a reduction in serotonin levels reduces the activity of serotonergic neurons and increases habituation process to acoustic stimulus (Pantoja et al., 2016). It is known that CAF reduces serotonin levels in the human brain (Lee and Kim, 2019) and in rats (Lim et al., 2001) supporting this hypothesis. A second hypothesis is related to the effects of CAF on learning. CAF can inhibit the activity of AChE in zebrafish larvae (De Farias et al., 2021), and this inhibition may occur through the non-selective antagonism of CAF with adenosine receptors, increasing the levels of available acetylcholine (Colovic et al., 2013), known to be an important neurotransmitter for good memory performance (Birks, 2006). CAF, like other A1 and A2A adenosine receptor antagonists, has been suggested to improve scopolamine-induced memory impairment. Zebrafish injected with 10,000 μg. kg^−1^ CAF for 2 h before being exposed to a 60,670 μg scopolamine solution showed robust memory retention in inhibitory avoidance task tests (Bortolotto et al., 2015). Therefore, CAF can act both on the serotonergic system, inducing habituation, and on the cholinergic system, inducing learning. However, future measurements of serotonin and acetylcholine levels can be carried out to better clarify these processes.

In the present study, the effects of CAF on zebrafish feeding behaviour, time spent in the low part of the aquarium in the new tank test, and duration of contact with the mirror appear to be dose-dependent. Low concentrations initially have a greater impact on fish social behaviour and may reduce the number of responses to sound. Thus, 0.5, and 1.5 μg. L^−1^ CAF, based on latency time and frequency of contact with the mirror, appear to increase the attentiveness and visual acuity of fish, increase aggressive behaviour and a decrease social behaviour. These concentrations moderately affected exploratory behaviour but exerted no effect on fish feeding behaviour. On the other hand, the highest concentration of CAF tested (300 μg. L^−1^) reduces exploratory behaviour (novel tank) and increases the latency time for the first contact with the mirror, indicating higher anxiety in the fish. The longer time for the first feeding approach and total food consumption may result from a change in exploratory behaviour, although other mechanisms may also be involved (e.g., reduced appetite and increased satiety hormones YY). Thus, the fish weight loss observed in this study may be attributed to alterations in feeding behaviour, as observed in this study, as well increased energy expenditure during CAF metabolism (Greenberg and Geliebter, 2012; Santos-Silva et al., 2018).

Data from the present study support the idea that CAF concentrations widely detected in the environment may have an impact on the behaviour of fish (e.g., feeding, aggressiveness and social behaviour) that may ultimately impact population growth.

## Conclusion

The effects of short-term exposure to environmental CAF concentrations were studied in adult zebrafish. Exposure to environmentally relevant concentrations of CAF-induced aggressive and anxious behaviour at all concentrations tested. The CAF-induced anxiety-like behaviours in zebrafish were characterised by a reduction in the exploratory ability and interest in food and increased aggressive behaviour. Low concentrations of CAF improved alertness and induce reduced sociability. In nature, the behavioural changes detected in this study suggest a potential to affect population growth and maintenance. Future work should focus on the mechanisms responsible for the behavioural changes detected in this study.

### Credit authorship contribution statement

N. Santos: conceptualization, methodology, writing—original draft, writing—review and editing; V. Picolo: methodology; I. Domingues: conceptualization, validation, writing—review and editing; V. Perillo: methodology; C.K.; R.A.R. Villacis: methodology; C.K. Grisolia: conceptualization, validation, review and editing; funding; M. Oliveira: conceptualization, validation, writing—review and editing. All authors have read and approved the final manuscript.

### Fundings

This work was supported by support of CESAM by FCT/MCTES (UIDP/50017/2020 + UIDB/50017/2020 + LA/P/0094/2020), through national funds, the PhD grant awarded to Niedja Santos (BD/REIT/8708/2019) and the Brazilian Research Council for Scientific and Technological Development (CNPq).

## Data Availability

The data are not shared but the data will be available if requested by the journal.
